# Di-μ-glutarato-κ^4^ *O* ^1^:*O* ^5^-bis­[aqua­(1,10-phenanthroline-κ^2^ *N*,*N*′)copper(II)]

**DOI:** 10.1107/S1600536811007938

**Published:** 2011-03-09

**Authors:** Yong-Hong Zhou

**Affiliations:** a School of Chemistry and Material Science, Huaibei Normal University, Huaibei 235000, People’s Republic of China

## Abstract

In the centrosymmetric dinuclear title complex, [Cu_2_(C_5_H_6_O_4_)_2_(C_12_H_18_N_2_)_2_(H_2_O)_2_], the Cu^II^ atom displays a dis­torted square-pyramidal coordination environment with the basal plane occupied by two phenanthroline N atoms and two O atoms from different glutarate dianions while a water mol­ecule is located at the apical position. Of the two water H atoms, one is engaged in an intra­molecular hydrogen bond with a free oxygen of the dianion whereas the second is engaged in an inter­molecular hydrogen bond, building a corrugated layer parallel to (100). These layers are further connected through π–π stacking inter­actions involving symmetry-related phenanthroline rings [centroid–centroid distance = 3.5599 (17) and 3.5617 (18) Å], building a three dimensionnal network. C—H⋯π inter­actions involving the phenanthroline ring system are also observed.

## Related literature

For coordination modes of the glutarate anion, see: Ghosh *et al.* (2007 ▶); Kim *et al.* (2005 ▶); Rather & Zaworotko (2003 ▶); Zheng *et al.* (2004 ▶); Vaidhyanathan *et al.* (2004 ▶); Girginova *et al.* (2007 ▶). 
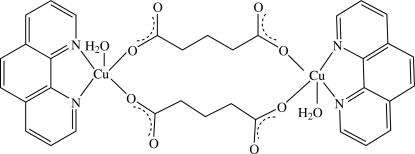


## Experimental

### 

#### Crystal data

[Cu_2_(C_5_H_6_O_4_)_2_(C_12_H_18_N_2_)_2_(H_2_O)_2_]
*M* *_r_* = 783.72
Monoclinic, 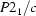

*a* = 10.2767 (11) Å
*b* = 10.5935 (14) Å
*c* = 15.5998 (16) Å
β = 107.114 (1)°
*V* = 1623.1 (3) Å^3^
*Z* = 2
Mo *K*α radiation
μ = 1.38 mm^−1^
*T* = 298 K
0.26 × 0.25 × 0.23 mm

#### Data collection

Bruker SMART CCD area-detector diffractometer
Absorption correction: multi-scan (*SADABS*; Bruker, 1997 ▶) *T*_min_ = 0.716, *T*_max_ = 0.742
7937 measured reflections
2867 independent reflections
2275 reflections with *I* > 2σ(*I*)
*R*_int_ = 0.028

#### Refinement

*R*[*F* ^2^ > 2σ(*F* ^2^)] = 0.031
*wR*(*F* ^2^) = 0.083
*S* = 1.07
2867 reflections
226 parameters
H-atom parameters constrained
Δρ_max_ = 0.31 e Å^−3^
Δρ_min_ = −0.28 e Å^−3^

### 

Data collection: *SMART* (Bruker, 1997 ▶); cell refinement: *SAINT* (Bruker, 1997 ▶); data reduction: *SAINT*; program(s) used to solve structure: *SHELXTL* (Sheldrick, 2008 ▶); program(s) used to refine structure: *SHELXL97* (Sheldrick, 2008 ▶); molecular graphics: *ORTEPIII* (Burnett & Johnson, 1996 ▶), *ORTEP-3 for Windows* (Farrugia, 1997 ▶) and *PLATON* (Spek, 2009 ▶); software used to prepare material for publication: *SHELXL97*.

## Supplementary Material

Crystal structure: contains datablocks I, New_Global_Publ_Block. DOI: 10.1107/S1600536811007938/dn2661sup1.cif

Structure factors: contains datablocks I. DOI: 10.1107/S1600536811007938/dn2661Isup2.hkl

Additional supplementary materials:  crystallographic information; 3D view; checkCIF report

## Figures and Tables

**Table 1:** Hydrogen-bond geometry (Å, °) *Cg*1 is the centroid of the N1,C6–C10 ring

*D*—H⋯*A*	*D*—H	H⋯*A*	*D*⋯*A*	*D*—H⋯*A*
O5—H51⋯O4	0.89	1.81	2.659 (3)	158
O5—H52⋯O2^i^	0.88	1.89	2.762 (3)	169
C2—H2*A*⋯*Cg*1^i^	0.97	2.88	3.754 (3)	151

Symmetry code: (i) 
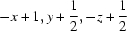
.

**Fig. 1.**
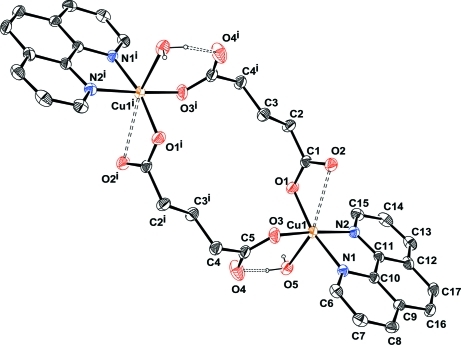
The molecular structure of the title compound with the atom labeling scheme. Displacement thermal paremeters are represented at the 30% probability level. Hydrogen bonds are shown as dashed lines. H atoms not involved in hydrogen bondings have been omitted for the sake of clarity. [Symmetry code: (i) -x + 1, -y + 1, -z + 1]

**Fig. 2.**
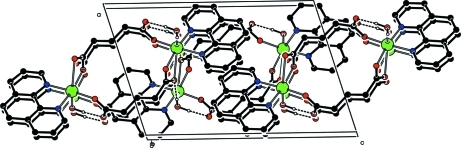
Partial packing view showing the formation of layer through O—H···O hydrogen bonds which are shown as dashed lines. H atoms not involved in hydrogen bondings have been omitted for the sake of clarity.

## supplementary crystallographic information

### Comment

For many yeras, there is a growing interest in developing organic-inorganic hybrid materials owing to their intriguing structures, new topologies, and potential applications(Ghosh *et al.*, 2007; Kim *et al.*,2005). Carboxylic acids have been proved to be versatile functional moieties in generating interesting hybrid materials by interacting with metal ions. The abilities of its anion to metal ions in diverse and unique linking modes can be regarded as a major factor in making the carboxylate function a versatile structure directing moiety.

Metal glutarates are one class of dicarboxylate system which exhibit interesting structural features. Previous investigations have demonstrated that glutaric acid presents interesting behaviors due to its conformational flexibility and coordination diversity (Rather *et al.*, 2003; Zheng *et al.*, 2004; Vaidhyanathan *et al.*, 2004; Girginova *et al.*, 2007). We report here the crystal structure of the title compound.

The title complex, [Cu(C_12_H_18_N_2_)(C_5_H_6_O_4_)(H_2_O)]_2_, is a dinuclear compound organized around inversion center. The Cu^II^ displays a distorted square pyramidal coordination environment (Fig. 1). The basal plane is occupied by two nitrogen atoms of the phenanthroline [Cu—N(1) = 2.014 (2)Å and Cu—N(2) = 2.022 (2) Å] and two O atoms from different glutarate dianions[Cu—O(1) = 1.954 (2)Å and Cu—O(3) = 1.947 (2) Å], whereas one water molecule is located at the apical position at a significantly longer distance[Cu—O(5) = 2.380 (2) Å]. The glutarate dianions act as a bidentate ligand bridging the two Cu^II^ ions which are separated by 8.476 Å.

There is an intramolecular hydrogen bond involving one H of the water and the O4 oxygen of one dianion within the dinuclear complex. The second H atom of the water is engaged in hydrogen bond interaction with the O2 oxygen atom of symmetry related dinuclear complex building then a corrugated layer parallel to the (1 0 0) plane (Fig. 2, Table 1). The layers are interconnected through π-π stacking involving the symmetry related N1,C6,C7,C8,C9,C10 (A) and N2,C11,C12,C13,C14,C15 (B) phenanthroline rings (Fig. 2, Table 2) building a three dimensional network. The packing is further stabilized by weak C—H···π interaction involving the symmetry related ring A (Table 1).

### Experimental

The title complex was prepared by the addition of the stoichiometric amount of CuCl_2_ (0.134 g, 1 mmol) to an ethanol solution of glutaric acid (0.264 g, 2 mmol) and 1,10-phenanthroline monohydrate(0.396 g, 2 mmol), the pH was adjusted to ~6 with 0.2 mol.*L*^-1^ KOH solution. The resulting solution was stirred for 30 min at room temperature and then filtered. Blue single crystals were isolated from the solution at room temperature over two weeks.

### Refinement

All H atoms attached to C atoms were fixed geometrically and treated as riding with C—H = 0.93 Å (aromatic) or 0.97 Å (methylene) with *U*_iso_(H) = 1.2*U*_eq_(C). H atoms of water molecule were located in difference Fourier maps and included in the subsequent refinement using restraints (O—H= 0.88 (1)Å and H···H= 1.50 (2) Å) with *U*_iso_(H) = 1.5*U*_eq_(O). In the last cycles of refinement, they were treated as riding on their parent O atoms.

### Crystal data

**Table:**

[Cu_2_(C_5_H_6_O_4_)_2_(C_12_H_18_N_2_)_2_(H_2_O)_2_]	*F*(000) = 804
*M**_r_* = 783.72	*D*_x_ = 1.604 Mg m^−^^3^
Monoclinic, *P*2_1_/*c*	Mo *K*α radiation, λ = 0.71073 Å
Hall symbol: -P 2ybc	Cell parameters from 3334 reflections
*a* = 10.2767 (11) Å	θ = 2.4–27.3°
*b* = 10.5935 (14) Å	µ = 1.38 mm^−^^1^
*c* = 15.5998 (16) Å	*T* = 298 K
β = 107.114 (1)°	Block, blue
*V* = 1623.1 (3) Å^3^	0.26 × 0.25 × 0.23 mm
*Z* = 2	

### Data collection

**Table:**

Bruker SMART CCD area-detector diffractometer	2867 independent reflections
Radiation source: fine-focus sealed tube	2275 reflections with *I* > 2σ(*I*)
graphite	*R*_int_ = 0.028
φ and ω scans	θ_max_ = 25.0°, θ_min_ = 2.4°
Absorption correction: multi-scan (*SADABS*; Bruker, 1997)	*h* = −11→12
*T*_min_ = 0.716, *T*_max_ = 0.742	*k* = −12→10
7937 measured reflections	*l* = −18→16

### Refinement

**Table:**

Refinement on *F*^2^	Primary atom site location: structure-invariant direct methods
Least-squares matrix: full	Secondary atom site location: difference Fourier map
*R*[*F*^2^ > 2σ(*F*^2^)] = 0.031	Hydrogen site location: inferred from neighbouring sites
*wR*(*F*^2^) = 0.083	H-atom parameters constrained
*S* = 1.07	*w* = 1/[σ^2^(*F*_o_^2^) + (0.0346*P*)^2^ + 1.103*P*] where *P* = (*F*_o_^2^ + 2*F*_c_^2^)/3
2867 reflections	(Δ/σ)_max_ < 0.001
226 parameters	Δρ_max_ = 0.31 e Å^−^^3^
0 restraints	Δρ_min_ = −0.28 e Å^−^^3^

### Special details

**Table:**

Geometry. All e.s.d.'s (except the e.s.d. in the dihedral angle between two l.s. planes) are estimated using the full covariance matrix. The cell e.s.d.'s are taken into account individually in the estimation of e.s.d.'s in distances, angles and torsion angles; correlations between e.s.d.'s in cell parameters are only used when they are defined by crystal symmetry. An approximate (isotropic) treatment of cell e.s.d.'s is used for estimating e.s.d.'s involving l.s. planes.
Refinement. Refinement of *F*^2^ against ALL reflections. The weighted *R*-factor *wR* and goodness of fit *S* are based on *F*^2^, conventional *R*-factors *R* are based on *F*, with *F* set to zero for negative *F*^2^. The threshold expression of *F*^2^ > σ(*F*^2^) is used only for calculating *R*-factors(gt) *etc*. and is not relevant to the choice of reflections for refinement. *R*-factors based on *F*^2^ are statistically about twice as large as those based on *F*, and *R*- factors based on ALL data will be even larger.

### Fractional atomic coordinates and isotropic or equivalent isotropic displacement parameters (Å^2^)

**Table:**

	*x*	*y*	*z*	*U*_iso_*/*U*_eq_	
Cu1	0.34382 (3)	0.54797 (3)	0.21857 (2)	0.03292 (13)	
N1	0.2087 (2)	0.4429 (2)	0.12652 (14)	0.0299 (5)	
N2	0.3977 (2)	0.6012 (2)	0.10928 (14)	0.0318 (5)	
O1	0.5068 (2)	0.61750 (19)	0.30440 (12)	0.0416 (5)	
O2	0.5850 (2)	0.43066 (19)	0.28151 (13)	0.0432 (5)	
O3	0.2798 (2)	0.4813 (2)	0.31524 (13)	0.0467 (5)	
O4	0.1462 (3)	0.6404 (2)	0.32731 (17)	0.0689 (7)	
O5	0.2215 (2)	0.74242 (19)	0.19357 (13)	0.0441 (5)	
H51	0.1900	0.7287	0.2401	0.066*	
H52	0.2746	0.8093	0.2014	0.066*	
C1	0.5979 (3)	0.5330 (3)	0.32214 (17)	0.0318 (6)	
C2	0.7228 (3)	0.5601 (3)	0.39982 (18)	0.0397 (7)	
H2A	0.7284	0.6499	0.4125	0.048*	
H2B	0.8038	0.5354	0.3841	0.048*	
C3	0.7158 (3)	0.4873 (3)	0.48313 (18)	0.0380 (7)	
H3A	0.6265	0.4993	0.4910	0.046*	
H3B	0.7266	0.3980	0.4735	0.046*	
C4	0.1767 (3)	0.4723 (3)	0.43155 (18)	0.0401 (7)	
H4A	0.1805	0.3816	0.4242	0.048*	
H4B	0.0869	0.4938	0.4356	0.048*	
C5	0.2012 (3)	0.5385 (3)	0.35137 (18)	0.0387 (7)	
C6	0.1142 (3)	0.3640 (3)	0.13755 (19)	0.0370 (7)	
H6	0.1091	0.3496	0.1953	0.044*	
C7	0.0230 (3)	0.3025 (3)	0.0664 (2)	0.0417 (7)	
H7	−0.0423	0.2487	0.0768	0.050*	
C8	0.0291 (3)	0.3208 (3)	−0.0187 (2)	0.0401 (7)	
H8	−0.0321	0.2800	−0.0667	0.048*	
C9	0.1287 (3)	0.4017 (3)	−0.03337 (18)	0.0339 (6)	
C10	0.2164 (3)	0.4611 (2)	0.04186 (17)	0.0292 (6)	
C11	0.3172 (3)	0.5485 (2)	0.03226 (17)	0.0294 (6)	
C12	0.3286 (3)	0.5760 (3)	−0.05311 (18)	0.0368 (7)	
C13	0.4278 (3)	0.6647 (3)	−0.0576 (2)	0.0445 (8)	
H13	0.4398	0.6864	−0.1126	0.053*	
C14	0.5061 (3)	0.7183 (3)	0.0194 (2)	0.0472 (8)	
H14	0.5717	0.7776	0.0171	0.057*	
C15	0.4890 (3)	0.6853 (3)	0.1024 (2)	0.0397 (7)	
H15	0.5435	0.7237	0.1542	0.048*	
C16	0.1444 (3)	0.4307 (3)	−0.11997 (19)	0.0429 (8)	
H16	0.0881	0.3915	−0.1707	0.051*	
C17	0.2384 (3)	0.5131 (3)	−0.12896 (19)	0.0453 (8)	
H17	0.2455	0.5299	−0.1859	0.054*	

### Atomic displacement parameters (Å^2^)

**Table:**

	*U*^11^	*U*^22^	*U*^33^	*U*^12^	*U*^13^	*U*^23^
Cu1	0.0360 (2)	0.0377 (2)	0.02556 (18)	0.00182 (16)	0.00982 (14)	−0.00183 (15)
N1	0.0338 (12)	0.0290 (12)	0.0281 (11)	0.0019 (10)	0.0112 (10)	0.0016 (9)
N2	0.0340 (13)	0.0280 (12)	0.0336 (12)	0.0029 (10)	0.0105 (10)	0.0010 (10)
O1	0.0444 (12)	0.0396 (12)	0.0349 (11)	0.0059 (10)	0.0024 (9)	−0.0066 (9)
O2	0.0467 (13)	0.0412 (12)	0.0381 (11)	0.0073 (10)	0.0072 (9)	−0.0055 (9)
O3	0.0591 (14)	0.0546 (14)	0.0325 (11)	0.0083 (11)	0.0230 (10)	0.0053 (10)
O4	0.095 (2)	0.0595 (16)	0.0710 (16)	0.0257 (15)	0.0535 (15)	0.0232 (14)
O5	0.0495 (12)	0.0443 (12)	0.0379 (11)	−0.0074 (10)	0.0120 (9)	−0.0047 (9)
C1	0.0351 (15)	0.0379 (17)	0.0232 (13)	−0.0013 (13)	0.0097 (11)	0.0041 (12)
C2	0.0365 (16)	0.0490 (18)	0.0310 (14)	−0.0073 (14)	0.0057 (12)	0.0045 (13)
C3	0.0428 (17)	0.0383 (17)	0.0316 (15)	−0.0051 (13)	0.0090 (13)	0.0043 (13)
C4	0.0410 (17)	0.0481 (19)	0.0319 (15)	−0.0069 (14)	0.0117 (13)	−0.0004 (13)
C5	0.0417 (17)	0.0492 (19)	0.0253 (14)	−0.0065 (15)	0.0100 (12)	−0.0020 (14)
C6	0.0390 (16)	0.0338 (16)	0.0417 (16)	0.0031 (13)	0.0174 (13)	0.0044 (13)
C7	0.0359 (16)	0.0317 (16)	0.0580 (19)	−0.0016 (13)	0.0147 (14)	−0.0013 (14)
C8	0.0325 (16)	0.0339 (16)	0.0475 (18)	0.0027 (13)	0.0019 (13)	−0.0101 (14)
C9	0.0342 (15)	0.0326 (15)	0.0313 (14)	0.0095 (12)	0.0041 (12)	−0.0017 (12)
C10	0.0319 (14)	0.0280 (14)	0.0274 (13)	0.0071 (12)	0.0084 (11)	0.0002 (11)
C11	0.0320 (14)	0.0287 (14)	0.0287 (14)	0.0089 (12)	0.0105 (11)	0.0026 (11)
C12	0.0443 (17)	0.0357 (16)	0.0342 (15)	0.0159 (13)	0.0177 (13)	0.0100 (12)
C13	0.0488 (19)	0.0447 (19)	0.0476 (18)	0.0138 (15)	0.0260 (15)	0.0163 (15)
C14	0.0453 (18)	0.0354 (17)	0.070 (2)	0.0027 (14)	0.0307 (17)	0.0137 (16)
C15	0.0370 (16)	0.0318 (16)	0.0503 (18)	0.0004 (13)	0.0131 (14)	−0.0021 (14)
C16	0.0475 (18)	0.0480 (19)	0.0289 (15)	0.0103 (15)	0.0046 (13)	−0.0058 (13)
C17	0.058 (2)	0.055 (2)	0.0246 (15)	0.0181 (17)	0.0136 (14)	0.0054 (14)

### Geometric parameters (Å, °)

**Table:**

Cu1—O3	1.947 (2)	C4—C3^i^	1.520 (4)
Cu1—O1	1.9545 (19)	C4—H4A	0.9700
Cu1—N1	2.014 (2)	C4—H4B	0.9700
Cu1—N2	2.022 (2)	C6—C7	1.387 (4)
Cu1—O5	2.385 (2)	C6—H6	0.9300
N1—C6	1.329 (3)	C7—C8	1.362 (4)
N1—C10	1.360 (3)	C7—H7	0.9300
N2—C15	1.320 (4)	C8—C9	1.404 (4)
N2—C11	1.362 (3)	C8—H8	0.9300
O1—C1	1.266 (3)	C9—C10	1.401 (4)
O2—C1	1.243 (3)	C9—C16	1.440 (4)
O3—C5	1.267 (3)	C10—C11	1.429 (4)
O4—C5	1.225 (4)	C11—C12	1.402 (4)
O5—H51	0.8897	C12—C13	1.403 (4)
O5—H52	0.8804	C12—C17	1.435 (4)
C1—C2	1.511 (4)	C13—C14	1.359 (4)
C2—C3	1.531 (4)	C13—H13	0.9300
C2—H2A	0.9700	C14—C15	1.401 (4)
C2—H2B	0.9700	C14—H14	0.9300
C3—C4^i^	1.520 (4)	C15—H15	0.9300
C3—H3A	0.9700	C16—C17	1.340 (5)
C3—H3B	0.9700	C16—H16	0.9300
C4—C5	1.518 (4)	C17—H17	0.9300
			
O3—Cu1—O1	91.32 (9)	H4A—C4—H4B	108.2
O3—Cu1—N1	91.85 (9)	O4—C5—O3	125.6 (3)
O1—Cu1—N1	165.65 (9)	O4—C5—C4	119.0 (3)
O3—Cu1—N2	173.39 (9)	O3—C5—C4	115.3 (3)
O1—Cu1—N2	94.62 (9)	N1—C6—C7	122.6 (3)
N1—Cu1—N2	81.69 (9)	N1—C6—H6	118.7
O3—Cu1—O5	99.10 (8)	C7—C6—H6	118.7
O1—Cu1—O5	95.20 (7)	C8—C7—C6	120.0 (3)
N1—Cu1—O5	98.12 (8)	C8—C7—H7	120.0
N2—Cu1—O5	83.28 (8)	C6—C7—H7	120.0
C6—N1—C10	117.9 (2)	C7—C8—C9	119.4 (3)
C6—N1—Cu1	129.16 (18)	C7—C8—H8	120.3
C10—N1—Cu1	112.87 (17)	C9—C8—H8	120.3
C15—N2—C11	117.8 (2)	C10—C9—C8	117.3 (3)
C15—N2—Cu1	129.3 (2)	C10—C9—C16	117.9 (3)
C11—N2—Cu1	112.56 (17)	C8—C9—C16	124.8 (3)
C1—O1—Cu1	108.23 (17)	N1—C10—C9	122.8 (2)
C5—O3—Cu1	125.2 (2)	N1—C10—C11	116.4 (2)
Cu1—O5—H51	91.5	C9—C10—C11	120.7 (2)
Cu1—O5—H52	113.4	N2—C11—C12	123.6 (3)
H51—O5—H52	112.2	N2—C11—C10	116.3 (2)
O2—C1—O1	123.0 (2)	C12—C11—C10	120.0 (2)
O2—C1—C2	120.8 (3)	C11—C12—C13	116.9 (3)
O1—C1—C2	116.1 (3)	C11—C12—C17	118.2 (3)
C1—C2—C3	110.1 (2)	C13—C12—C17	124.9 (3)
C1—C2—H2A	109.6	C14—C13—C12	119.0 (3)
C3—C2—H2A	109.6	C14—C13—H13	120.5
C1—C2—H2B	109.6	C12—C13—H13	120.5
C3—C2—H2B	109.6	C13—C14—C15	120.7 (3)
H2A—C2—H2B	108.1	C13—C14—H14	119.6
C4^i^—C3—C2	113.5 (2)	C15—C14—H14	119.6
C4^i^—C3—H3A	108.9	N2—C15—C14	121.9 (3)
C2—C3—H3A	108.9	N2—C15—H15	119.1
C4^i^—C3—H3B	108.9	C14—C15—H15	119.1
C2—C3—H3B	108.9	C17—C16—C9	121.4 (3)
H3A—C3—H3B	107.7	C17—C16—H16	119.3
C5—C4—C3^i^	109.7 (2)	C9—C16—H16	119.3
C5—C4—H4A	109.7	C16—C17—C12	121.8 (3)
C3^i^—C4—H4A	109.7	C16—C17—H17	119.1
C5—C4—H4B	109.7	C12—C17—H17	119.1
C3^i^—C4—H4B	109.7		

Symmetry codes: (i) −*x*+1, −*y*+1, −*z*+1.

### Hydrogen-bond geometry (Å, °)

**Table:**

Cg1 is the centroid of the N1,C6–C10 ring

**Table:**

*D*—H···*A*	*D*—H	H···*A*	*D*···*A*	*D*—H···*A*
O5—H51···O4	0.89	1.81	2.659 (3)	158
O5—H52···O2^ii^	0.88	1.89	2.762 (3)	169
C2—H2A···Cg1^ii^	0.97	2.88	3.754 (3)	151

Symmetry codes: (ii) −*x*+1, *y*+1/2, −*z*+1/2.

### Table 2 Table 2 π-π stacking interactions (Å)

Cg1 is the centroid of the N1,C6–C10 ring. Cg2 is the centroid of the N2,C11–C15 ring

**Table:**

CgI	CgJ	centroid-to-centroid	interplanar vector	Slippage
Cg1	Cg1^ii^	3.5599 (17)	3.342	1.226
Cg2	Cg2^iii^	3.5617 (18)	3.374	1.142

Symmetry codes: (ii)-x,1-y,1-z; (iii) 1-x, 1-y, -zSlippage = vertical displacement between ring centroids.
